# Optimized method for polarization-based image dehazing

**DOI:** 10.1016/j.heliyon.2023.e15849

**Published:** 2023-04-28

**Authors:** Chunsheng Sun, Zhichao Ding, Liheng Ma

**Affiliations:** College of Ordnance Engineering, Naval University of Engineering, Wuhan 430033, China

**Keywords:** Image dehazing, Combined dehazing method, Polarization-based dehazing, Contrast-enhancement-based dehazing

## Abstract

Image dehazing is desired under the foggy, rainy weather, or the underwater condition. Since the polarization-based image dehazing utilizes additional polarization information of light to de-scatter, image detail can be recovered well, but how to segment the polarization information of the background radiance and the object radiance becomes the key problem. For solving this problem, a method which combing polarization and contrast enhancement is demonstrated. This method contains two main steps, (a) by seeking the region of large mean intensity, low contrast and large mean degree of polarization, the no-object region can be found, and (b) through defining a weight function and judging whether the dehazed image can achieve high contrast and low information loss, the degree of polarization for object radiance can be estimated. Based on the estimated parameters, the scatter of light by the mediums can be diminished considerably. The theoretical derivation shows that this method can achieve advantages complementation, such as being able to obtain more details like the polarization-based method and high image contrast like the contrast enhancement based method. Besides, it is physically sound and can achieve good dehazing performance under different conditions, which has been verified by different hazing polarization images.

## 1 Introduction

Under the pursue of a high degree of automation, target recognition, location and tracking based on image information have been attracting wide attention. In these applications, for achieving high performances, images with high contrast and definition are desired. In the clear sky, the desired images can be obtained simply. However, in some cases such as the foggy or rainy weather, or the underwater condition, the quality of images degrades to a great degree due to the strong absorption and scattering of light by the mediums [1]. Since these situations are common in practical applications, improving the quality of images under these situations, commonly referred to as the image dehazing, becomes a focus of current research [2].

In order to realize the image dehazing, various of methods have been proposed, which can be broadly categorized as polarization-based [[3], [4], [5]], filtering-based [6,7], dark channel prior based [8,9], contrast enhancement based [10,11], fusion-based [1,12], and machine learning based image dehazing (14; 15). All these methods have their own characteristics. For instances, the algorithm of filtering-based image dehazing is simple, but for the image of low contrast, the effect of dehazing is not obvious. The dark channel prior based image dehazing is based on the laws of physics, and can obtain good performance for most hazy images, but under the dense foggy weather or underwater condition, it usually cannot achieve the desired results. In addition, the fusion-based image dehazing with complex algorithm can improve the quality of almost all the hazy images, and the machine learning based image dehazing is a new emerging method but has some problems to be solved.

For the contrast enhancement based image dehazing, the key operation is to maximize the contrast for the whole image or each dividing region of image by histogram equalization, Bi-histogram modification, transmittance optimization, or some other ways [2,10,11]. The contrast enhancement based image dehazing can improve the contrast of almost all the hazy images, however, it is easy to cause the distortion of images. For the polarization-based image dehazing, the polarization information of light, which is not considered by other methods, is acquired by a polarization imaging sensor. Since the polarization information of the background radiance and the object radiance is different, the image can be dehazed by segmenting the object and background relying to the gathered polarization information. Since the polarization-based image dehazing utilizes more information of light, it has many advantages such as the simple algorithm and the ability of dehazing under the dense foggy weather, but how to segment the polarization information of the background radiance and the object radiance is the key problem of this dehazing method. One segmentation approach is that the degree of polarization (DoP) of the object radiance is assumed to be zero [3]. However, it will lead to some errors for this assumption especially when the object is smooth like the lake. So, the DoP of the object radiance had better be considered, though it adds the difficulty of the segmentation of the polarization information. For this reason, some prior attempts to evaluate the DoPs of the background radiance and the object radiance are reported, such as assuming that the background radiance and the object radiance are uncorrelated [4], or the object radiance and the medium transmittance are uncorrelated [13].

As can be seen from the above common methods of polarization-based image dehazing, an assumption is needed for evaluating the DoPs of the background radiance and the object radiance. However, the above assumptions are built on the empirical law and not physically sound. In addition, so far, there is no attempt to combine the polarization-based and other methods of image dehazing. So, if we can realize a method which is physically sound by combing the polarization-based method and another dehazing method, it may achieve the advantages complementation and make the dehazing effect better than that achieved by a separate one, and obtain excellent dehazing performance for different degrading images. Considering that a reasonable evaluation of the DoPs of the background radiance and the object radiance will lead to a high contrast of image, we can use contrast-enhancement-based method to assist the polarization-based method. Besides, the polarization-based method usually will not cause the distortion of images, which can make up the limitation of contrast-enhancement-based method.

Given all this, in this paper, we demonstrate a new method for polarization-based image dehazing, which is referred to as the CPCE (polarization and contrast enhancement) method. The theoretical principle is analyzed through a step-by-step derivation, and the dehazing performance is verified by experiment. This method combines polarization-based and contrast enhancement based methods. It is physically sound, and can achieve good dehazing performance under different conditions.

## 2 Method

For the optical imaging of an object, the information of the object observed by the eyes or cameras is modulated on the optical signal, and the optical signal stems from the reflection of the natural or artificial light by the object. As depicted in Ref. [3], under the foggy or rainy weather, or the underwater condition, the observed intensity image *I* can be given by

(1) $$I=D+A=Lt+A_{0}\left(1-t\right)\text{.}$$

Here, *L* represents the irradiance reflected by the object, *D* is the transmitted part of *L* after *L* passing through the medium between the object and observer, *A* represents the background radiance, in other words, the backscatter caused by the medium, *A*_0_ is the value of *A* when there is no object in the line of sight (LOS), and *t* is the transmittance of the medium, which can be derived as

(2) $$t=1-\frac{A}{A_{0}}\text{.}$$

Among the parameters in Eq. (1), *I* is the observed intensity image and can be directly obtained from the acquired polarization image *I*_p_, and *L* is the desired object information. According to Eqs. (1), (2), in order to extract *L* from *I*, at least two of the three parameters *A*_0_, *t* and *A* should be obtained.

### 2.1 Derivation of background radiance

Since *A*_0_ is equal to *I* where there is no object in the LOS, it can be approximately regarded as a global constant and obtained by extracting the non-object points in the observed intensity image *I* [3,4]. The non-object points have many characteristics such as high intensity, low contrast and high DoP [3,10]. For this reason, many approaches can be used to derive *A*_0_. For instances, in Ref. [7], *A*_0_ is set as the intensity of 0.1% times the brightest pixel in *I*. In Ref. [8], the 0.1% brightest pixels in the dark channel is picked, and *A*_0_ is set as the highest intensity of these picked pixels in *I*. Besides, in Ref. [10], the region with the maximum value of the mean intensity subtracting the contrast is selected, and *A*_0_ is set as the intensity of the brightest pixel in the selected region.

For the above approaches, the non-object region in *I* is regarded to possess highest intensity, lowest contrast, or highest intensity in the dark channel. Though these assumptions are rational in most situations, there are some errors in some special cases such as when a high reflectivity or uniform object exists in the LOS. In view of this, for minimizing the errors of searching the non-object points in these special cases, a weight function $W_{\Omega }^{A_{0}}$ is defined to estimate *A*_0_ as below

(3) $$W_{\Omega }^{A_{0}}=\sum_{c\in \left\{r,g,b\right\}}\left({\bar{I}}_{\Omega }^{c}+k_{1}C_{\Omega }^{c}+k_{2}{\bar{P}}_{\Omega }^{c}\right)\text{.}$$

Here, ${\bar{I}}_{\Omega }^{c}$, $C_{\Omega }^{c}$, and ${\bar{P}}_{\Omega }^{c}$ represent the mean intensity, the contrast, and the mean DoP for a color channel c∈{r,g,b} of a region Ω in a RGB image, respectively, and $C_{\Omega }^{c}$ is given by

(4) $$C_{\Omega }^{c}={\sum_{\Omega }\left[I_{\Omega }^{c}\left(x\right)-{\bar{I}}_{\Omega }^{c}\right]}^{2}/N_{\Omega }^{c}\text{.}$$

In Eq. (4), $I_{\Omega }^{c}\left(x\right)$ is the intensity for a color channel of a pixel *x* in Ω, and $N_{\Omega }^{c}$ is the number of pixels in Ω. *k*_1_ and *k*_2_ are two positive weight factors. The option of *k*_1_ and *k*_2_ is based on the prior characters of the object and background. For example, if we know the object is uniform, a small *k*_1_ (<1, such as 0.5) should be set.

In order to derive *A*_0_, the acquired polarization image is divided into small regions with the same size of $m_{0}\times n_{0}$ ($m_{0}=16$ and $n_{0}=16$ are chosen in this paper) firstly. Then, $W_{\Omega }^{A_{0}}$ is calculated based on Eq. (3) for each region, and the region $\Omega_{m}$ with maximum $W_{\Omega }^{A_{0}}$ is picked since the non-object region has the characteristics of high mean intensity, contrast, and mean DoP. Finally, *A*_0_ is estimated by

(5) $${\tilde{A}}_{0}^{c}={\bar{I}}_{\Omega_{m}}^{c}\;\left(\Omega_{m}=\arg\max_{\Omega \in I_{P}}\left\{W_{\Omega }^{A_{0}}\right\}\right)\text{.}$$

Here, ${\tilde{A}}_{0}^{c}$ is the estimated value of *A*_0_ in a color channel. After calculating ${\tilde{A}}_{0}^{c}$ for all the color channels based on Eq. (5), the estimated value ${\tilde{A}}_{0}$ of *A*_0_ is obtained.

### 2.2 Derivation of transmittance

For the polarization-based image dehazing, *t* is estimated by deriving *A* first. As demonstrated in Ref. [4], *A* can be derived from the polarization information as below

(6) $$A=I\frac{P-P_{D}}{P_{A}-P_{D}}\text{,}$$

where $P_{A}$, $P_{D}$ and *P* are the DoPs of the background radiance *A*, object radiance *D* and observed intensity image *I*, respectively. Since *I* and *P* can be directly obtained from the acquired polarization image [14], $P_{A}$ and $P_{D}$ should be known if we want to get *A*.

As $P_{A}$ is equal to *P* where there is no object in the LOS, it can be approximately regarded as a global constant and obtained by extracting the DoP in the non-object region [3,4]. The non-object region has already been obtained when deriving *A*_0_, which is actually $\Omega_{m}$. So, $P_{A}$ is estimated by

(7) $${\tilde{P}}_{A}^{c}={\bar{P}}_{\Omega_{m}}^{c}\;\left(\Omega_{m}=\arg\max_{\Omega \in I_{P}}\left\{W_{\Omega }^{A_{0}}\right\}\right)\text{.}$$

Here, ${\tilde{P}}_{A}^{c}$ is the estimated value of $P_{A}$ in a color channel. After calculating ${\tilde{P}}_{A}^{c}$ for all the color channels based on Eq. (7), the estimated value ${\tilde{P}}_{A}$ of $P_{A}$ is obtained.

From the above analysis, we can find that the key of polarization-based image dehazing is the derivation of $P_{D}$. For achieving this goal, several different schemes have been proposed. For examples, in Ref. [3], $P_{D}$ is assumed to be zero, and in Refs. [4,13], $P_{D}$ is derived by assuming that *A* and *D* are uncorrelated [4], or the object information *L* and the transmittance *t* are uncorrelated [13]. These are traditional polarization-based methods. In Section 1, we have demonstrated the advantages and disadvantages of these schemes. Here, we will show our scheme as below.

In order to derive $P_{D}$, the acquired polarization image is divided into small regions with the same size of $m_{0}\times n_{0}$ ($m_{0}=16$ and $n_{0}=16$ are chosen in this paper). Since in a small region, the variation of the object is not obvious, $P_{D}$ is approximately regarded to be unchanged and can be derived separately for each region.

According to Eqs. (1), (6), when $P_{D}$ is set as a series of values, different results of *t* and *L* can be obtained. As the haze degrades the contrast of the observed image, the dehazed image is expected to have a high contrast. Therefore, we can pick $P_{D}$ which yields a high contrast of *L*. Even so, it can be observed that when $P_{D}$ is set as some values, the intensities of some pixels in *L* are not in the valid range of 0 to 255, and the values of *t* for some pixels in *L* are not in the valid range of 0 to 1, which will causing the information loss of the dehazed image and the estimation error of *t*. Considering the above situations, a weight function $W_{\Omega }^{P_{D}}$ is defined to estimate $P_{D}$ in each region,

(8) $$\left\{ \begin{array}{l} W_{\Omega }^{P_{D}}=W_{C}^{c}-q_{1}W_{L}^{c}-q_{2}W_{E}^{c}\\ W_{C}^{c}={\sum_{\Omega }\left[L_{\Omega }^{c}\left(x\right)-{\bar{L}}_{\Omega }^{c}\right]}^{2}/N_{\Omega }^{c}\\ W_{L}^{c}=\sum_{\Omega }\left\{{\left[\min\left\{0,L_{\Omega }^{c}\left(x\right)\right\}\right]}^{2}+{\left[\max\left\{0,L_{\Omega }^{c}\left(x\right)-255\right\}\right]}^{2}\right\}/N_{\Omega }^{c}\\ W_{E}^{c}=\sum_{\Omega }\left\{{\left[\min\left\{0,t_{\Omega }^{c}\right\}\right]}^{2}+{\left[\max\left\{0,t_{\Omega }^{c}-1\right\}\right]}^{2}\right\}/N_{\Omega }^{c} \end{array}\right.\text{.}$$

Here, $L_{\Omega }^{c}$, ${\bar{L}}_{\Omega }^{c}$, and $t_{\Omega }^{c}$ are the calculated intensity of *L*, mean intensity of *L*, and *t* for a color channel of a region Ω when $P_{D}$ is set as a certain value, respectively. $W_{C}^{c}$, $W_{L}^{c}$, and $W_{E}^{c}$ are the weight subfunctions, respectively reflecting the contrast of $L_{\Omega }^{c}$, the information loss of the dehazed image, and the error of $t_{\Omega }^{c}$. $q_{1}$ and $q_{2}$ are two positive weight factors, and the option of $q_{1}$ and $q_{2}$ is based on the prior characters of the acquired polarization image.

As can be seen from Eq. (8), a high contrast of $L_{\Omega }^{c}$, a small information loss of the dehazed image, as well as a small error of $t_{\Omega }^{c}$ will yield a large $W_{\Omega }^{P_{D}}$. Therefore, $P_{D}$ can be estimated by

(9) $${\tilde{P}}_{D,\Omega }^{c}=\arg\max_{P_{D}\in \left\{0,P_{0}\right\}}\left\{W_{\Omega }^{P_{D}}\right\}\text{,}$$

where ${\tilde{P}}_{D,\Omega }^{c}$ is the estimated value of $P_{D}$ in a color channel of the region Ω, and $P_{0}$ is a constant representing the maximum value possible of $P_{D}$, which can be set as the maximum value of *P*. For the estimation in Eq. (9), $P_{D}$ is set as a series of values, and then $W_{\Omega }^{P_{D}}$ is calculated. An optimal value ${\tilde{P}}_{D,\Omega }^{c}$ of $P_{D}$ is determined by finding the maximum $W_{\Omega }^{P_{D}}$. After estimating ${\tilde{P}}_{D,\Omega }^{c}$ for all the color channels and the regions using the above method, the estimated value ${\tilde{P}}_{D}$ of $P_{D}$ is obtained.

When $P_{D}$ is obtained, using the polarization-based method as shown in Eqs. (6), (7), image dehazing can be realized. In addition, according to Eqs. (8), (9), the estimated value of $P_{D}$ will lead to a high contrast of the dehazed image. So, in this stage the contrast-enhancement-based concept is integrated in the polarization-based dehazing. For this reason, polarization and contrast enhancement is combined for the proposed method.

### 2.3 Recovery of the haze image

After extracting *I* and *P* from the acquired polarization image, and estimating $P_{A}$ and $P_{D}$ using the above method, the estimated value of *A* can be derived based on Eq. (6). Substituting *A* and *A*_0_ into Eq. (2), the estimated value of *t* is obtained. Since the above calculation is built on the image region, *t* will show block artifacts. For solve this problem, *t* is usually refined [9]. Here, we choose the guided filter to refine *t*, the detail algorithm is shown in Ref. [15], and the result of *t* after guided filter is represented as $\hat{t}$.

After obtaining *A*_0_ and $\hat{t}$, the dehazed image *L* can be given based on Eq. (1)

(10) $$\tilde{L}=\frac{I-{\tilde{A}}_{0}}{\max\left(\hat{t},t_{0}\right)}+{\tilde{A}}_{0}\text{.}$$

Here, *t*_0_ is used to avoid a low value of the denominator causing excessive large noise, 0.1 for example [9].

Finally, for diminishing color distortion, white balancing with the well-knowing Gray-World algorithm is adopted for foggy or rainy images, and underwater white balancing with the algorithm proposed by Ancuti et al. is adopted for underwater images [16].

**Fig. 1:**
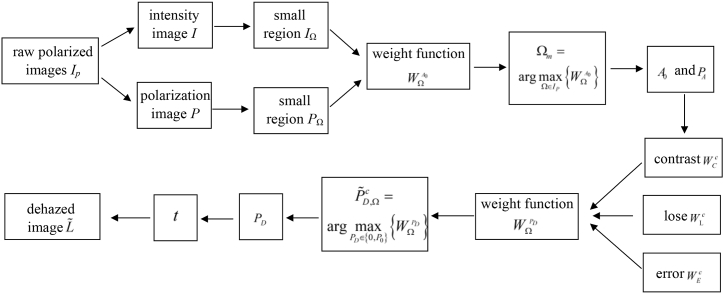
Steps of the CPCE method.

Fig. 1 summarizes the steps of our CPCE method. Firstly, gather the raw polarized image *I*_p_, and extract the intensity image *I* and polarization image *P* from *I*_p_. Secondly, divide *I* and *P* into small region *I*_Ω_ and *P*_Ω_, respectively, and determine *A*_0_ and *P*_A_ using the weight function $W_{\Omega }^{A_{D}}$. Then, based on the contrast subfunction $W_{C}^{c}$, the loss subfunction $W_{L}^{c}$, and the error subfunction $W_{E}^{c}$, determine *P*_*D*_ using the weight function $W_{\Omega }^{P_{D}}$. Finally, calculate *A* and *t* using the estimated value of *P*_A_ and *P*_*D*_, and restore the dehazed image *L* according to Eq. (10).

## 3 Evaluation

**Fig. 2:**
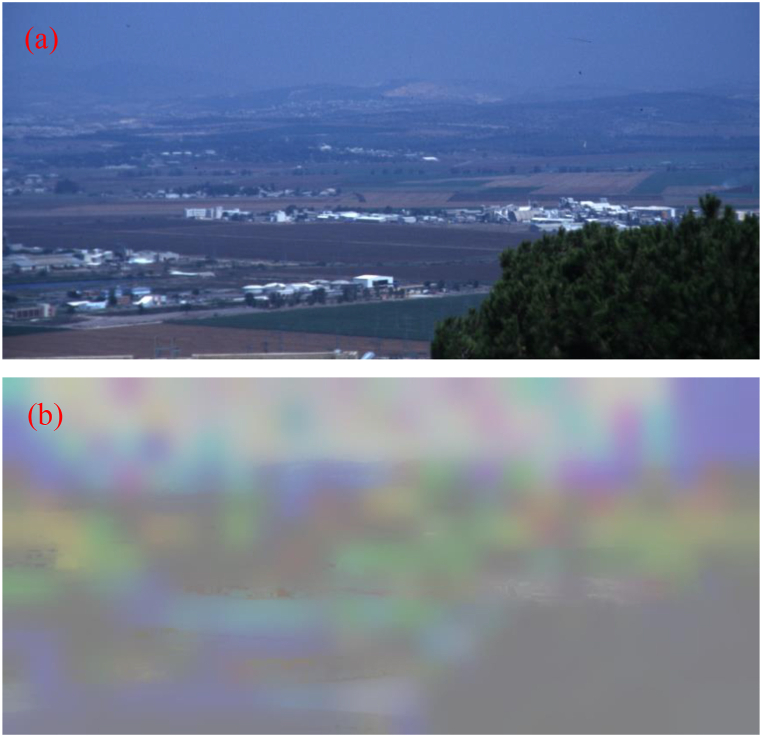
Classic foggy scene. (a) intensity image *I*; (b) object radiance image ${\hat{P}}_{D}+0.5$.

**Fig. 3:**
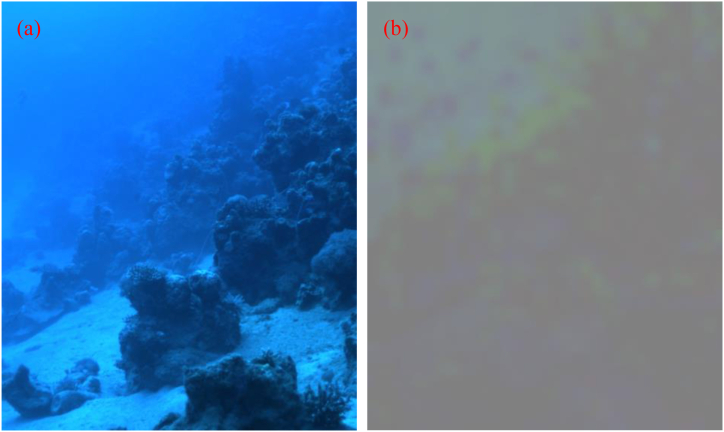
Classic underwater scene. (a) intensity image *I*; (b) object radiance image ${\hat{P}}_{D}+0.5$.

In order to evaluate the efficiency of the CPCE method, a classic foggy scene with the size of 871 ${}^{*}$ 411*3 and a classic underwater scene with the size of 505*609*3 captured by Schechner et al. are used [3,17]. For either scene, images of the worst polarization state and the best polarization state are acquired, respectively. By comparing the two images of orthogonal polarization states, *I* and *P* can be extracted as demonstrated in Ref. [3]. Fig. 2, Fig. 3, which show obvious degrading of image quality, are the intensity images for the foggy and underwater scenes, respectively.

As demonstrated in the above part, we do not assume that the DoP of the object radiance is equal to zero, and $P_{D}$ is estimated using the above method. For clearly showing the estimated value of $P_{D}$, we choose the guided filter to refine $P_{D}$, and the results ${\hat{P}}_{D}$ of guided filter plus 0.5 are shown in Fig. 2, Fig. 3.

From Fig. 2, Fig. 3, we can find that after our estimation, the DoP of the object radiance is no longer zero, and varies with the sight. Comparing Fig. 2(a) and (b), we can find that for the smooth places such as sky, field and lake, ${\hat{P}}_{D}$ is relatively large, while for the rough objects like trees and buildings, ${\hat{P}}_{D}$ is relatively small. This tendency can also be seen in Fig. 3(b). The season for this tendency is mainly that the light incident on smooth places is mostly reflected not scattered, and can maintain a large DoP. So, the estimated result of $P_{D}$ is rational.

**Fig. 4:**
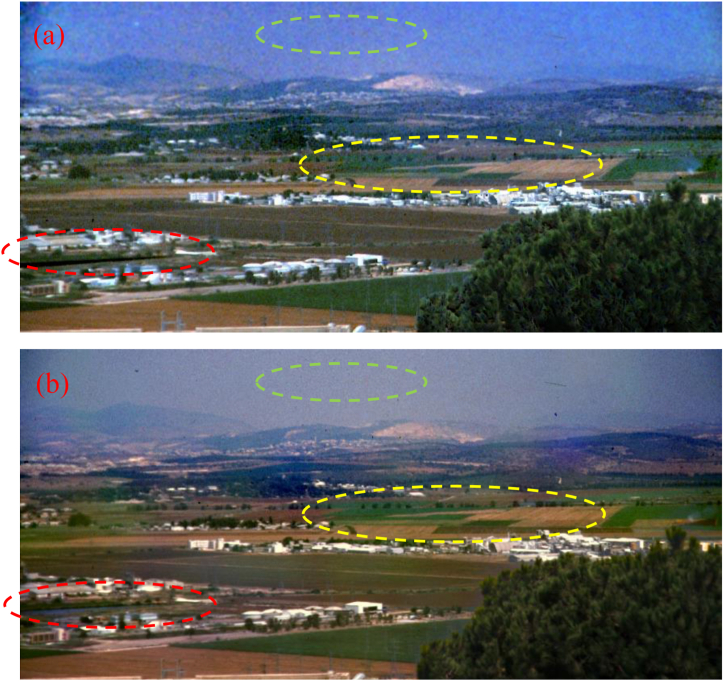
Dehazing results for the foggy scene. (a) Schechner’ method; (b) the CPCE method.

**Fig. 5:**
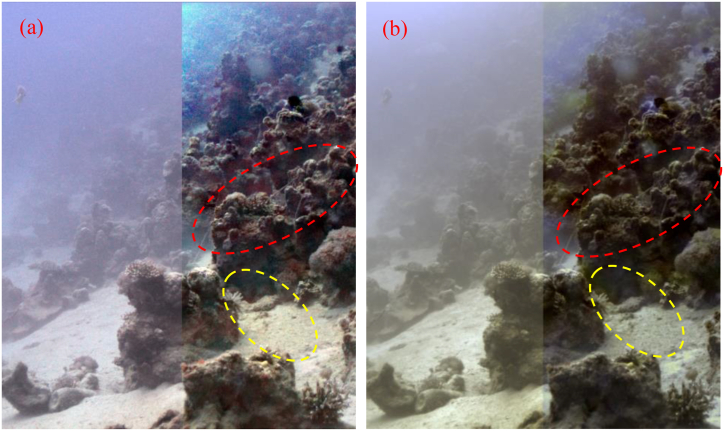
Dehazing results for the underwater scene. (a) Schechner’ method; (b) the CPCE method. The left parts in these two images are the naive white-balancing results.

Fig. 4, Fig. 5 show the dehazed images using the method proposed by Schechner et al. when assuming that the background radiance and the object radiance are uncorrelated, and the estimated result of $P_{D}$ is zero [3,4,17]. The dehazing results by Fang et al. can be seen in Ref. [13]. Fig. 4, Fig. 5 are the dehazing results using the CPCE method with $q_{1}=5$, $q_{2}=50$, and $t_{0}=0.3$. Comparing the dehazing results using different methods, we can find that though all the methods can realize good image dehazing, using the CPCE method, the dehazed images are finer, and the noise is relatively low especially for the smooth areas like sky and field, verifying the superiority of the CPCE method.

## 4 Experiment

**Fig. 6:**
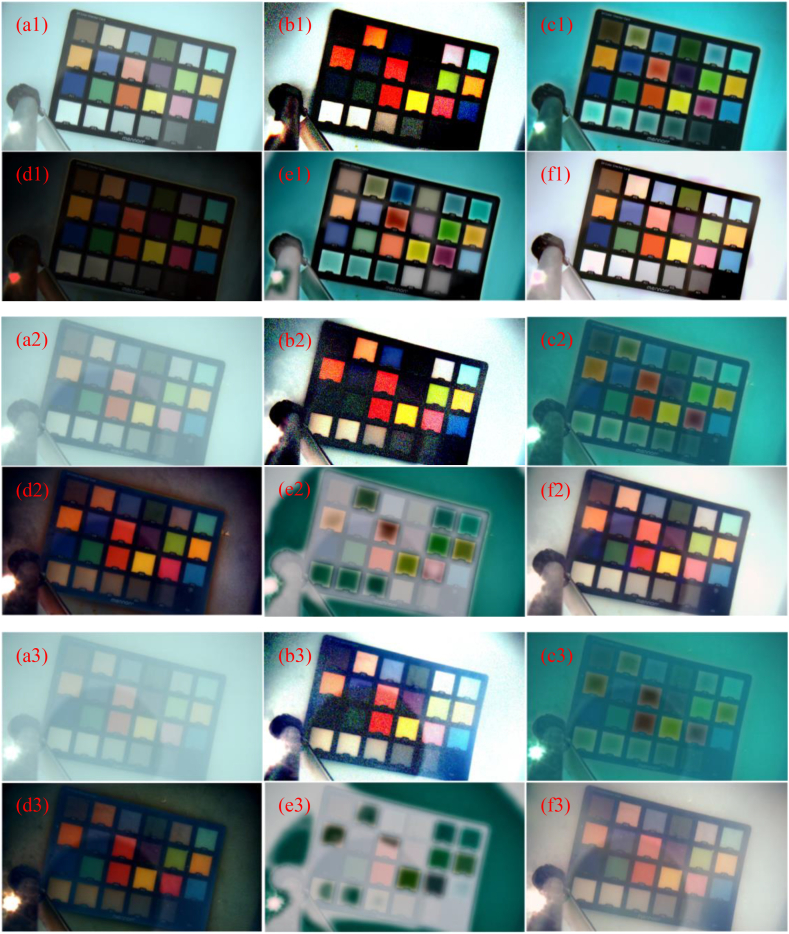
Dehazing results for captured scenes. (a1) - (a3): original scenes; (b1) - (b3): Schechner’ method; (c1) - (c3): He’ method; (d1) – (d3): Fattal’ method; (e1) – (e3): Ancuti’ method; (f1) –(f3): the CPCE method.

In order to further verify the efficiency of the CPCE method, we have captured some underwater images. A 24 color checker card produced by Mennon of China is placed in the water with a depth of about 0.5 m, and a division-of-focal-plane (DoFP) polarimeter BFS–U3–51S5PC-C manufactured by Flir Systems Inc. of USA on the water is adapted to acquire polarization images. From the polarization images acquired by the DoFP polarimeter, we can extract the intensity image and the polarization image [18]. Fig. 6(a1)-6 (a3) show three intensity images captured by our experimental setup. For Fig. 6(a2) and 6 (a3), some milks are added into the water, and the addition level for Fig. 6(a3) is larger than that for Fig. 6(a2).

Some commonly used methods are selected to dehaze the images of Fig. 6(a1) - (a3). Fig. 6(b1) - 6 (b3) show the dehazed images using the Schechner's method proposed by Ref. [4], Fig. 6(c1) - 6 (c3) show the dehazed images using the He's method proposed by Ref. [8], Fig. 6(d1) - 6 (d3) show the dehazed images using the Fattal's method proposed by Ref. [19], and Fig. 6(e1) - 6 (e3) show the dehazed images using the Ancuti's method proposed by Ref. [12]. Fig. 6(f1) - 6 (f3) are the dehazing results using the CPCE method with $q_{1}=5$, $q_{2}=50$, and $t_{0}=0.3$. Comparing the dehazing results using different methods, we can find that in the underwater condition, since the strong scattering exists, the methods without using the polarization information of light are easy to cause the color distortion of images. In addition, Schechner's method can diminish scatter surrounding the object to a certain extent, however, it brings considerable noise and causes overexpose, and the dehazing effects for the background region is not obvious. While using the CPCE method, the dehazed images are finer, the noise is relatively low, and image dehazing is effective not only in the region of the object, but also in the region of background, verifying the efficiency of the CPCE method.

For comparing the dehazing efficiencies of different methods objectively, we calculate the peak signal-to-noise ratio (PSNR) and structural similarity index measurement (SSIM) [20] of the dehazing images in Fig. 6 comparing to the corresponding hazing images, and the results are shown in Table 1. We can find that among the above five kinds of methods, the proposed CPCE method can achieve the highest PNSR and SSIM, further verifying the efficiency of the CPCE method.

**Table 1:** PSNR and SSIM of the dehazed images using different methods.

Method	PNSR SSIM (comparing to Fig (a1))	PNSR SSIM (comparing to Fig (a2))	PNSR SSIM (comparing to Fig (a3))
Schechner’	42.3 0.986	49.6 0.978	54.8 0.987
He’	59.2 0.990	57.2 0.983	56.8 0.981
Fattal’	52.7 0.951	51.2 0.955	51.3 0.949
Ancuti’	56.8 0.984	56.4 0.981	56.4 0.980
CPCE	**65.00.998**	**60.80.993**	**63.30.996**

## 5 Conclusions

In summary, for improving the quality of images captured in foggy or rainy weather, or the underwater condition, we have demonstrated a method combing polarization and contrast enhancement to decrease the influence caused by the scattering of light by the mediums. In order to find the no-object region and extract the intensity and DoP of this region, the region of large mean intensity, low contrast and large mean DoP is seeked. More importantly, this method considers the DoP of object radiance, and a weight function is defined to estimate the DoP of object radiance by judging whether can achieve high contrast and low information loss of the dehazed image. According to the estimated DoP of object radiance, and the derived intensity and DoP of the no-object background radiance, the scatter of light by the mediums can be diminished considerably, and the dehazed image can be obtained. Using the classic hazing polarization images and the images captured by the experiment, we have demonstrated that the proposed method can achieve good dehazing performance under different conditions.

Since this method combines polarization-based and contrast enhancement based methods, it is physically sound, and is applicable to different hazing images. However, the set of initial parameters relies on the size and quality of the hazing images for the proposed method, which is not automatic and needs further study to consummate.

## Declarations

### Author contribution statement

Zhichao Ding: Conceived and designed the experiments; Performed the experiments; Analyzed and interpreted the data; Contributed reagents, materials, analysis tools or data; Wrote the paper.

Chunsheng Sun: Conceived and designed the experiments; Performed the experiments.

Liheng Ma: Analyzed and interpreted the data.

### Funding statement

Zhichao Ding was supported by National Natural Science Foundation of China [62205375].

### Data availability statement

Data will be made available on request.

### Additional information

No additional information is available for this paper.

## Declaration of competing interest

The authors declare that they have no known competing financial interests or personal relationships that could have appeared to influence the work reported in this paper.
